# Real-world treatment patterns and outcomes in chronic lymphocytic leukemia: a multicenter retrospective study in Greece

**DOI:** 10.1007/s00277-026-07161-3

**Published:** 2026-07-03

**Authors:** Epameinondas Koumpis, Eva Minga, Persefoni Talimtzi, Maria Angelopoulou, Sofia Chatzileontiadou, Maria Dimou, Sotiria Besikli-Dimou, Maria Efstathopoulou, Dimitris Gogos, Evdoxia Hatjiharissi, Eleftheria Hatzimichael, Michail Iskas, Eliana Konstantinou, Ioannis Kotsianidis, Alexandros Machairas, Eugenia Mpalaoura, Anastasia Mpanti, Panagiotis Panagiotidis, Gerasimos Pangalis, Ioannis Papadopoulos, Maria Papaioannou, Marina Siakantaris, Niki Stavroyianni, Theodoros Vassilakopoulos, George Vrachiolias, Kostas Stamatopoulos, Anastasia Chatzidimitriou, Thomas Chatzikonstantinou

**Affiliations:** 1 Department of Haematology, Faculty of Medicine, University of Ioannina, Greece, Ioannina, Greece; 2 Institute of Applied Biosciences, Center for Research and Technology, Thessaloniki, Greece; 3 Haematology, University of Athens, Laikon General Hospital, Athens, Greece; 4 1st Dept of Internal Medicine, Hematology Unit, AUTH, AHEPA Hospital, Thessaloniki, Greece; 5 Hematology Department and HCT Unit G. Papanicolaou Hospital Thessaloniki, Thessaloniki, Greece; 6 Department of Haematology, Athens Medical Center, Athens, Greece; 7 Hospital of Mytilene, Mytilene, Greece; 8 Department of Hematology, University Hospital of Alexandroupolis, Democritus University of Thrace, Alexandroupolis, Greece; 9 Department of Hematology, Papageorgiou Hospital, Thessaloniki, Greece

**Keywords:** CLL, Treatment patterns, Greece, Targeted agents

## Abstract

**Purpose:**

This study aims to characterize treatment patterns for chronic lymphocytic leukemia (CLL) in real-world clinical practice across Greece focusing on both treatment-naïve and relapsed or refractory (R/R) disease. We evaluated therapeutic patterns and outcomes [including overall survival (OS), time to next treatment or death (TTNT-D) and overall response rate (ORR)] and documented the incidence of other malignancies of patients who initiated first-line treatment between 2010 and 2024.

**Methods:**

This is a panhellenic multicenter retrospective study of patients diagnosed with CLL between 2010 and 2020 from 8 centers in Greece.

**Results:**

Overall, 1318 patients were included in the study. Fludarabine, cyclophosphamide and rituximab (FCR) was the most common first-line regimen (21.6%), followed by chlorambucil plus rituximab (16.3%) and ibrutinib-based therapies (14%). In the R/R setting, ibrutinib and venetoclax-based treatments, became the predominant therapeutic choices after 2014, replacing chemoimmunotherapy. Shifts in treatment patterns before and after 2016 reflected the incorporation of targeted agents into routine clinical practice, with a marked decline in FCR use and increased adoption of targeted therapies. Median OS was 162 months from diagnosis and 105.2 months (95% CI: 90.3–116.3) from first treatment. In patients receiving ibrutinib as first-line therapy, mOS was not reached, and median TTNT-D was 67.3 months.

Overall, 18 (1.4%) cases developed a second hematological malignancy and 59 (4.5%) were diagnosed with a solid tumor. The most common hematological malignancy was myelodysplastic neoplasms and the most common solid tumors included lung, skin, breast, colon and prostate cancers.

**Conclusion:**

The findings suggest a gradual shift in CLL treatment patterns in Greece, with targeted agents increasingly replacing traditional chemoimmunotherapy in recent years and showing favorable survival outcomes.

**Supplementary Information:**

The online version contains supplementary material available at https://doi.org/10.1007/s00277-026-07161-3.

## Introduction

New small molecule inhibitors have transformed the therapeutic landscape of chronic lymphocytic leukemia (CLL). Notably, Bruton tyrosine kinase inhibitor (BTKi) and BCL2 inhibitor-based therapies have consistently demonstrated superior efficacy compared to chemoimmunotherapy (CIT) in both treatment-naïve and relapsed/refractory (R/R) settings [1].

We conducted this panhellenic multicenter retrospective study aiming to (i) describe treatment patterns in real-world clinical practice in Greece, (ii) assess the therapeutic outcomes of patients who received first-line treatment between 2010 and 2024 and (iii) document the occurrence of other malignancies within this patient population.

## Methods

Investigators from participating sites contributed data from patients with CLL. All human participants included in this study provided informed consent prior to their participation. The study has been approved by the ethics and research integrity committee of the Centre for Research and Technology Hellas and conducted according to the Declaration of Helsinki.

Data were collected on patients diagnosed with CLL between 2010 and 2020, within the study period spanning from 2010 to 2024. Clinical and laboratory data, treatments and outcomes were recorded for all patients. Treatment initiation was defined as the date of administration of the first dose of the respective CLL-directed therapy Patients were consecutively included at each center. Cases with missing information on the type of treatment, dates of diagnosis and first line treatment were excluded. Missing data were not imputed and the frequency of missingness is reported for all variables (supplemental Table 1). We utilized pairwise deletion for the univariate analysis and listwise deletion for the multivariable models.

**Table 1:** Reasons for Discontinuation of First-Line Ibrutinib Therapy

Adverse Event/Reason for Discontinuation	n	%
Cardiovascular events	5	23.8%
Rash	3	14.3%
Infections	2	9.5%
Pneumonitis	1	4.8%
Encephalitis	1	4.8%
Anemia	1	4.8%
Not specified/missing	8	38.1%
Total	21	100%

Median and interquartile range (IQR) were used to describe numeric variables, while frequencies and percentages were used for categorical. The significance level was set to 5%, and Wald’s confidence intervals were constructed. All statistical analyses were conducted in R (version 4.1.1), using the “survival” package for the Cox regression, and the “survminer” package for Kaplan–Meier visualization [2, 3]. Overall survival (OS) was defined as the time from CLL-directed treatment to death (event) or the last follow-up date (censoring). Time to next treatment or death (TTNT-D) was defined as the time from the start of the first CLL-directed treatment to initiating the next line of therapy or death (events) or the last follow-up date (censoring).

## Results/discussion

### Demographic and clinical characteristics

We collected data on 1318 patients with CLL from 8 centers in Greece. Most patients were male (810, 61.5%) and had at least one comorbidity at the time of diagnosis (1030, 82.2%). The median age at diagnosis was 65 years (IQR: 58–74) and the median age at first treatment was 68 years (IQR: 60–75). The median follow‐up from CLL diagnosis was 96.1 months (IQR 93–101). Immunoglobulin heavy variable (IGHV) gene somatic hypermutation status was assessed in 815 patients, with 430 (52.8%) and 385 (47.2%) having mutated and unmutated IGHV genes, respectively. *TP53* mutation status was assessed in 515 patients, with mutations identified in 50 cases (9.7%). Deletion 17p [del(17p)] was evaluated by fluorescence in situ hybridization (FISH) in 338 patients and detected in 31 cases (9.2%). Conventional karyotyping was performed in 661 patients, revealing abnormal karyotypes in 303 (45.8%) cases. Specifically, karyotypes with one or two aberrations were observed in 223 cases (33.7%), while karyotypes with three or four aberrations were detected in 51 (7.7%) cases, respectively. Finally, five or more aberrations were detected in 29 cases (4.4%) (7/29 had concurrent *TP53* aberrations). Almost half of the patients (686/1318, 52%) received at least one line of treatment, while the remaining 48% of patients remained untreated. Among treated patients, 377/686 (54.9%) received one line of treatment, 192 (28%) underwent two lines of treatment and 117 (17.1%) received 3 or more lines of treatment.

### Treatment patterns

The most common first‐line regimen was fludarabine, cyclophosphamide and rituximab (FCR) (148, 21.6%), followed by chlorambucil plus rituximab (112, 16.3%) and ibrutinib ± anti-CD20-antibody (96, 14%). Frontline regimens based on venetoclax were administered to 49 (7.1%) patients, of whom 40 received a combination of venetoclax plus obinutuzumab. The majority of patients with R/R disease received novel small molecule inhibitors in the second-line setting (203/309, 65.7%). The most common second line treatment regimens were ibrutinib (± anti CD20) (87, 28.2%) and venetoclax either as a monotherapy (27, 8.7%) or in combination with rituximab (56, 18.1%). Treatment beyond the second line was also predominantly based on novel small molecule inhibitors (predominantly regimens based on ibrutinib, acalabrutinib, venetoclax, and less frequently idelalisib) [84/117 (71.8%) in third line and 26/43 (60.5%) in fourth line].

**Fig. 1:**
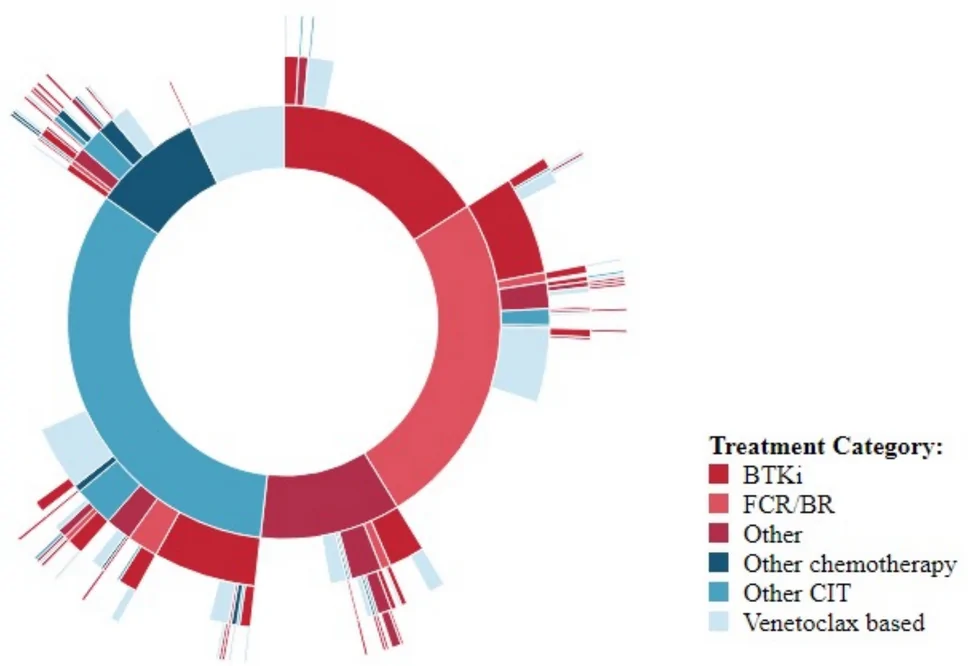
Sunburst diagram of treatment patterns. Each color indicates a particular treatment regimen, and each concentric layer represents a sequential line of therapy, starting with the first line at the center and progressing to the fourth line at the periphery. The white part in each line of therapy (concentric layer) represents patients who did not receive a new treatment line either due to continued response to the prior treatment or death

**Fig. 2:**
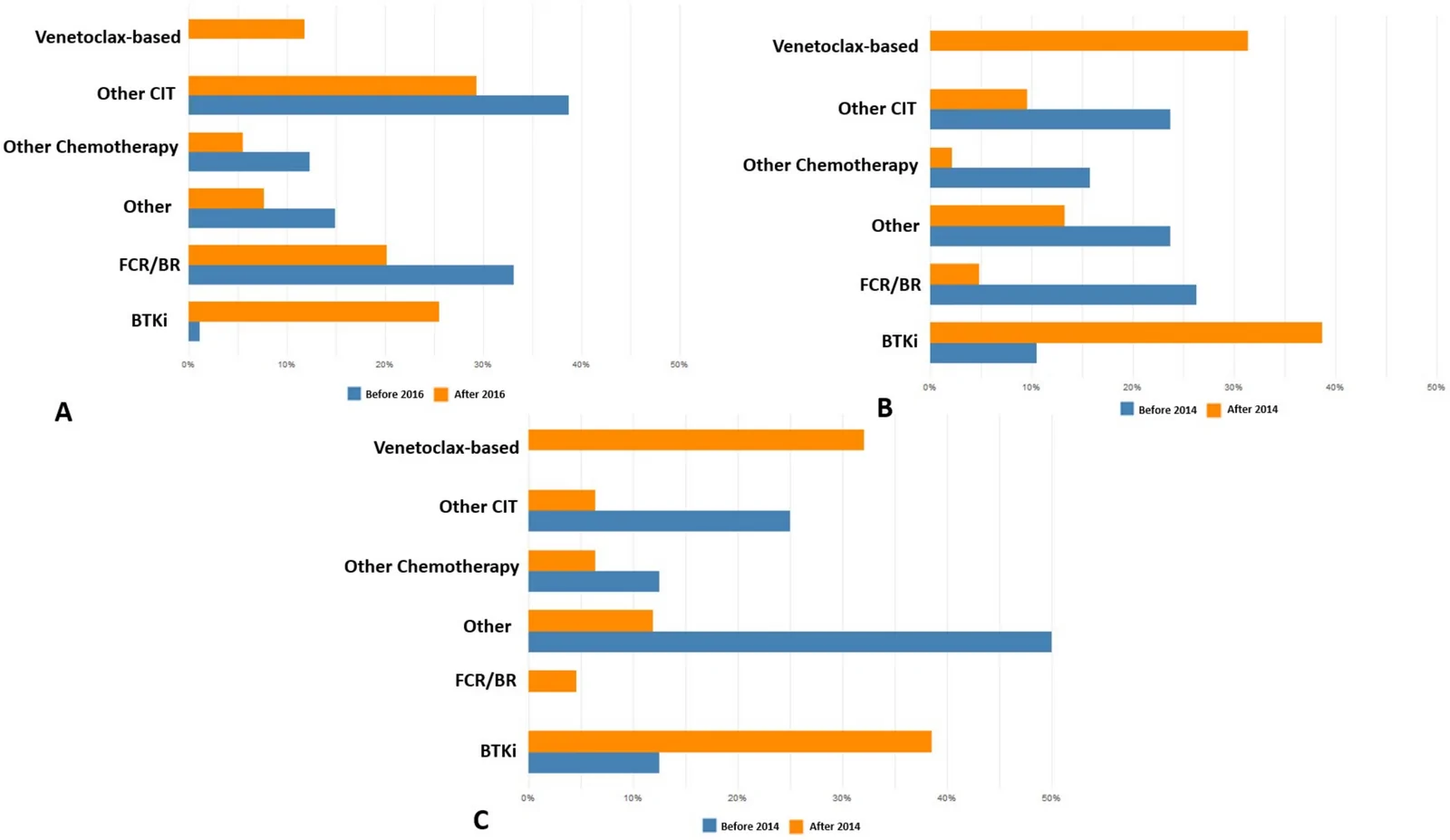
Barplots representing the percentage of patients treated with different regimens. (**A**) in first line, before and after 2016, (**B**) in second line, before and after 2014 and (**C**) in third line before and after 2014. Graph A illustrates the marked shift in first-line treatment patterns, with Fludarabine-Cyclophosphamide-Rituximab/Bendamustine-Rituximab predominating before 2016, whereas BTK inhibitors became the most commonly used regimen after 2016, accompanied by the introduction of venetoclax-based therapies. Similarly, graphs B and C demonstrate that chemoimmunotherapy was the predominant treatment approach in the second- and third-line settings before 2014, while BTK inhibitors and venetoclax-based regimens accounted for a substantial proportion of treatments following their introduction into clinical practice after 2014

Historically, CIT formed the foundation of CLL management, with regimens like FCR demonstrating durable responses, particularly in younger, fit patients with favorable genetic profiles [4]. Over the past decade, the therapeutic approach has undergone a significant shift with the introduction and widespread adoption of targeted agents [5–8]. This therapeutic shift was also noticed in our study (Fig. 1). Notably, before 2014, FCR was the predominant second-line treatment, administered to 21.1% of cases. However, following the European Medicines Agency (EMA) approval of ibrutinib for previously treated CLL in 2014, the use of FCR in the second-line setting declined markedly to 1.1%. During this period, most patients received ibrutinib (30.6%), venetoclax plus rituximab (20.7%), or venetoclax monotherapy (10%). Similarly, after the EMA approval of ibrutinib for first-line treatment in 2016, treatment patterns shifted substantially, with ibrutinib becoming the most frequently used first-line therapy (22.4% from 1.1% before 2016), while FCR use declined from 32% to 14.9%. The use of chlorambucil monotherapy or in combination with either rituximab or ofatumumab in first line declined after 2016 (9.3% to 5.1%, 20.8% to 13.5%, and 5.9% to 0.2%, respectively) while the use of chlorambucil plus obinutuzumab and bendamustine plus rituximab increased after 2016 (2.6% to 13.2% and 1.1% to 5.3%, respectively). Venetoclax plus obinutuzumab emerged as a first line treatment option in 9.6% of cases after 2016, aligning with its more recent approval [7, 9]. (Fig. 2).

### Other malignancies

Patients with CLL are at an increased risk of developing other malignancies (OMs), compared to the general population, a phenomenon likely attributable to both CLL-associated immune dysregulation and the effects of therapy [10]. Among the study population, 228 patients were diagnosed with at least one additional malignant neoplasm, with 154 of these malignancies occurring after the diagnosis of CLL. Of these 154 patients, 48 had not received treatment for CLL, whereas 106 had been treated. Among the treated patients who developed a subsequent malignancy, 81 had received chemotherapy-based regimens. In our study, among treated patients, 18 (1.4%) cases were diagnosed with hematological OMs [excluding Richter syndrome (RT)], 29 (2.2%) with RT and 59 (4.5%) solid tumors. OMs were diagnosed prior to the initiation of CLL treatment in 58.3%. The most common hematological OM was myelodysplastic neoplasms (3, 3.9% of OMs) and the most common solid tumors included lung cancer (10, 13% of OMs), nonmelanoma skin cancer (9, 11.7%), breast cancer (8, 10.4%), colon cancer (7, 9.1%), and prostate cancer (5, 6.5%). These findings are consistent with previous studies investigating OMs in CLL [10].

### TTFT, OS and TTNT-D

**Fig. 3:**
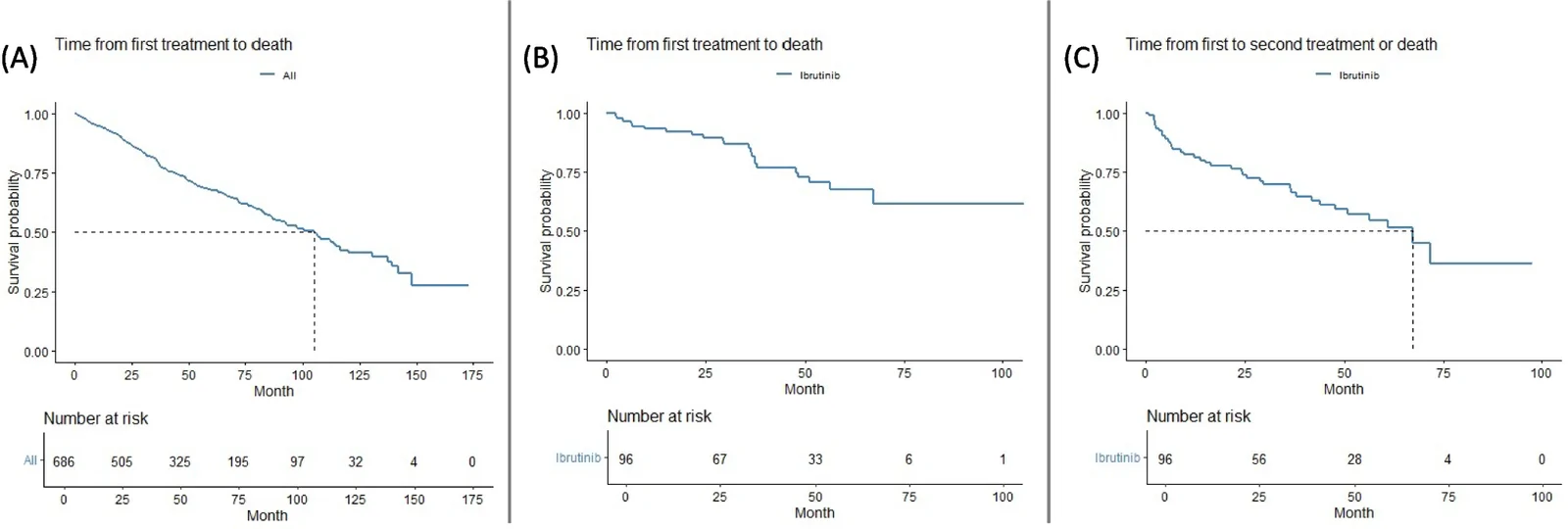
Overall survival (OS) from initiation of first treatment for all patients (median OS 105.2 months) (**A**). OS for patients treated with ibrutinib (median OS not reached) (**B**). Time to next treatment or death (TTNT-D) for patients treated with ibrutinib (median TTNT-D was 67.3 months) (**C**)

In our study, the median time from diagnosis of CLL to the first treatment (TTFT) was 65.4 months (95% CI = 59–76.4), reflecting the indolent nature of CLL in an unselected cohort. Similar to previously reported studies, the median overall survival (mOS) from diagnosis and first treatment were 162 [95% CI = 142-not estimable (NE)] and 105.2 months (95% CI = 90.3–116.3), respectively [1, 11, 12]. For cases treated with first-line ibrutinib, mOS was not reached at the time of analysis, and the median TTNT-D was 67.3 months (47.7-NE) (Fig. 3).

Venetoclax-based treatments have demonstrated high efficacy in the frontline treatment of CLL. However, mirroring their more recent approval, their use remained relatively limited in our study [9]. The ORR was 100% among patients who received the obinutuzumab–venetoclax and 91.7% for both obinutuzumab–chlorambucil and FCR treated patients. Furthermore, the TTNT-D was not reached for the obinutuzumab–venetoclax group, whereas it was 46 (95% CI: 36.9–59.6) and 58.4 months (95% CI: 43.2–74.3) for obinutuzumab–chlorambucil and FCR regimens, respectively. Of note, these comparisons are descriptive in nature and were not adjusted for potential confounding clinical and biological prognostic factors. Therefore, the observed differences should be interpreted with caution and should not be considered evidence of an independent prognostic effect.

### Risk factors for OS

Univariate and multivariate analyses were conducted to identify clinical and biological factors (assessed at first-line treatment initiation) associated with OS. In the univariate analysis, advanced age [HR 1.07 (1.06, 1.09), p < 0.001], male sex [HR 1.34 (1.02–1.76), p:0.035], chemotherapy (without immunotherapy) [HR 1.8 (1.05–3.08), p:0.033] and the presence of comorbidities at first-line treatment initiation [HR 2.22 (1.46–3.36), p < 0.001] were significantly associated with inferior OS. The median OS was 79.2 months for patients harboring *TP53* mutations compared with 112.3 months for those without *TP53* mutations, showing a trend toward inferior survival that did not reach statistical significance (p = 0.068). Patients with mutated IGHV gene status had a median OS of 114.5 months, whereas those with unmutated IGHV status had a median OS of 97.8 months; however, this difference was not statistically significant (p = 0.30). In the multivariate analysis, advanced age [HR 1.05 (1.02–1.08), p < 0.001] and unmutated IGHV genes [HR 1.82 (1.16, 2.87), p < 0.010] were the only independent predictors of inferior OS (supplemental Table 2).

### TTNT-D in specific subgroups

The impact of *TP53* and IGHV mutational status on TTNT-D was also evaluated. Patients harboring *TP53* mutations had a significantly shorter median TTNT-D compared with those without *TP53* mutations (26.1 months [95% CI, 16.1–42.0] vs. 42.7 months [95% CI, 39.6–50.4]; p < 0.001). Similarly, patients with unmutated IGHV genes exhibited a significantly shorter median TTNT-D than those with mutated IGHV genes (37.2 months [95% CI, 32.9–42.0] vs. 70.4 months [95% CI, 55.0–97.2]; p < 0.001). Among the different treatment groups, the median TTNT-D was 67.3 months (95% CI, 47.7–NE) for BTK inhibitors (BTKi), 52.5 months (95% CI, 42.6–64.2) for FCR/Bendamustine-Rituximab, and 35.4 months (95% CI, 31.1–40.7) for other CIT regimens. The median TTNT-D was not reached in patients treated with venetoclax-based regimens; however, follow-up in this subgroup was substantially shorter, limiting the interpretation of this finding. In the multivariable analysis, only unmutated IGHV genes (HR: 1.82, 95% CI: 1.16–2.87, p = 0.01) and older age (HR: 1.05, 95% CI: 1.02–1.08, p < 0.001) were associated with inferior TTNT-D (supplemental Table 3).

### Outcomes of patients treated with ibrutinib

Ibrutinib, the first-in-class BTKi, has been available and extensively used in both treatment-naïve and R/R settings, resulting in broader clinical experience and more comprehensive real-world data regarding its effectiveness and safety [13]. In our cohort, 96 patients received ibrutinib as first-line treatment. The ORR of this cohort was 95.5% (84/88), despite being enriched with cases with unmutated IGHV genes [66/80, 82.5%] and *TP53* aberrations [14/80, 17.5%].

Of the 96 patients who received ibrutinib as frontline therapy, 33 (34.4%) discontinued treatment. The primary reason for discontinuation was toxicity, accounting for 21 patients (21/96, 21.9%), while CLL progression and Richter’s transformation occurred in 4 (4/96, 4.2%) and 2 (2/96, 2.1%) patients, respectively. The most common reason for ibrutinib discontinuation was cardiovascular events (5/21, 23.8%). Additional adverse events contributing to treatment discontinuation included rash (3/21, 14.3%), followed by infections (2/21, 9.5%) pneumonitis (1/21, 4.8%), encephalitis (1/21, 4.8%), and anemia (1/21, 4.8%). (Table 1). The observed pattern of treatment discontinuation is consistent with previously published studies reporting cardiovascular toxicity as a major cause of ibrutinib discontinuation [14]*.* Overall, 22/96 (22.9%) patients died and only 2 (2.1%) deaths were related to CLL progression.

Ibrutinib was administered to 123 patients with R/R CLL, primarily as a second-line therapy (87/123 cases, 70.7%). High ORR were observed in patients treated with ibrutinib in R/R disease, with rates of 90.5%, 86.2% and 85.7% in the second, third, and in fourth line setting. Regarding the second line of treatment, treatment was discontinued in 45/87 cases (51.7%). The primary reasons for discontinuation of ibrutinib included toxicity (13, 14.9%), disease progression (12, 13.8%) and treatment-unrelated deaths (9, 10.3%). Similar to previous studies, ibrutinib-related toxicity was the leading cause of treatment discontinuation in our study [13]; nevertheless, clinical outcomes remained favorable in both treatment-naïve and R/R CLL [13].

### Limitations

We acknowledge certain limitations in our study. The retrospective nature of the study introduces the potential for missing data, sampling and attrition bias. To address these limitations, data were collected from consecutively treated patients at each participating center, and the analysis focused on robust and objective clinical endpoints, including TTNT and OS. In addition, the non-randomized nature of treatment allocation may have introduced confounding by indication. Follow-up duration varied among patients, reflecting the real-world setting of the study. Response assessments were performed according to routine clinical practice and were not centrally reviewed. Finally, the multicenter design may have introduced inter-center heterogeneity in patient management and follow-up procedures.

## Conclusion

In conclusion, we underscore the evolving treatment landscape of CLL in Greece, mirroring the global transition from conventional CIT to targeted therapies. Ibrutinib was widely adopted after 2016, demonstrating favorable long-term clinical outcomes. Finally, the notable incidence of OMs among patients with CLL highlights the need for sustained surveillance and a holistic approach to disease management, while it remains to be determined how incidence rates may evolve in the chemotherapy-free era.

## Supplementary Information

Below is the link to the electronic supplementary material.

Supplementary file 1 (DOCX 24 KB)

## Data Availability

The data supporting the findings of this study are available from the corresponding author upon reasonable request.

## Declarations

All human participants included in this study provided informed consent prior to their participation.

The study has been approved by the ethics and research integrity committee of the Centre for Research and Technology Hellas.
