# Supplementary material for: Real-world treatment patterns and outcomes in chronic lymphocytic leukemia: a multicenter retrospective study in Greece
Source: Ann Hematol. 2026 Jul 3;105(10):426. doi: 10.1007/s00277-026-07161-3 (PMC13597568; doi:10.1007/s00277-026-07161-3)
Supplement: Supplementary file 1 [file 277_2026_7161_MOESM1_ESM.docx]

| **Variable** | **Category** | **N** | **%** | **Missing, n (%)** |
| --- | --- | --- | --- | --- |
| **Sex** | Male | 810 | 61.5% | 0 |
|  | Female | 508 | 38.5% |  |
| **Comorbidity status** | Known (assessed) | 1253 | 95.1% | 65 (4.9%) |
|  | — Present | 1030 | 82.2%* |  |
|  | — Absent | 223 | 17.8%* |  |
| **IGHV gene mutational status** | Assessed | 815 | 61.8% | 503 (38.2%) |
|  | — Mutated | 430 | 52.8%* |  |
|  | — Unmutated | 385 | 47.2%* |  |
| ***TP53* mutation status** | Assessed | 515 | 39.1% | 803 (60.9%) |
|  | — Mutated | 50 | 9.7%* |  |
|  | — Wild-type | 465 | 90.3%* |  |
| **del(17p) by FISH** | Evaluated | 338 | 25.6% | 980 (74.4%) |
|  | — Positive | 31 | 9.2%* |  |
|  | — Negative | 307 | 90.8%* |  |
| **Conventional karyotyping** | Performed | 661 | 50.2% | 657 (49.8%) |
|  | — Abnormal | 303 | 45.8%* |  |
|  | — Normal | 358 | 54.2%* |  |
| **Abnormal karyotype subgroups** | 1–2 aberrations | 223 | 33.7%* |  |
|  | 3–4 aberrations | 51 | 7.7%* |  |
|  | ≥5 aberrations | 29 | 4.4%* |  |

**Supplemental Material**

**Supplemental table 1.** Baseline Characteristics and Missing Data (N=1318)

IGHV: Immunoglobulin heavy variable

** Percentage calculated relative to the assessed/evaluated subgroup denominator, not the total cohort (N = 1318).*

**Supplemental Table 2.** Univariate and multivariable analyses for overall survival

|  | **Univariate Cox** | | | | **Multivariate Cox** | | |
| --- | --- | --- | --- | --- | --- | --- | --- |
|  | **N** | **HR** | **95% CI** | **p-value** | **HR** | **95% CI** | **p-value** |
| **Age at first line** | 686 | 1.07 | 1.06, 1.09 | <0.001 | 1.05 | 1.02-1.08 | <0.001 |
| **Sex** | 686 |  |  |  |  |  |  |
| F |  | Ref. |  |  | Ref. |  |  |
| M |  | 1.34 | 1.02, 1.76 | 0.035 | 1.15 | 0.77, 1.70 | 0.5 |
| ***TP53* aberration** | 528 |  |  |  |  |  |  |
| No |  | Ref. |  |  | Ref. |  |  |
| Yes |  | 1.44 | 0.98, 2.12 | 0.067 | 1.41 | 0.86, 2.29 | 0.2 |
| **IGHV gene status** | 492 |  |  |  |  |  |  |
| Mutated |  | Ref. |  |  | Ref. |  |  |
| Unmutated |  | 1.18 | 0.85, 1.63 | 0.3 | 1.82 | 1.16, 2.87 | 0.010 |
| **Treatment categories** | 686 |  |  |  |  |  |  |
| BTKi |  | Ref. |  |  | Ref. |  |  |
| FCR/BR |  | 0.64 | 0.39, 1.06 | 0.085 | 1.08 | 0.54, 2.15 | 0.8 |
| Other |  | 1.02 | 0.58, 1.77 | >0.9 | 0.83 | 0.34, 2.08 | 0.7 |
| Other chemotherapy |  | 1.80 | 1.05, 3.08 | 0.033 | 0.84 | 0.33, 2.11 | 0.7 |
| Other CIT |  | 1.40 | 0.89, 2.21 | 0.14 | 1.06 | 0.54, 2.06 | 0.9 |
| Venetoclax-based |  | 0.50 | 0.15, 1.67 | 0.3 | 0.27 | 0.03, 2.06 | 0.2 |
| **Comorbidities at 1st-line treatment** | 609 |  |  |  |  |  |  |
| NO |  | Ref. |  |  | Ref. |  |  |
| YES |  | 2.22 | 1.46, 3.36 | <0.001 | 1.63 | 0.86, 3.08 | 0.13 |

CI = Confidence Interval, HR = Hazard Ratio, Ref. = reference category IGHV: Immunoglobulin heavy variable, BTKi: BTK inhibitor, FCR/BR: fludarabine, cyclophosphamide and rituximab/ bendamustine plus rituximab, CIT: chemoimmunotherapy

**Supplemental Table 3.** Univariate and multivariable analyses for time to next treatment or death.

|  | **Univariate Cox** | | | | **Multivariate Cox** | | |
| --- | --- | --- | --- | --- | --- | --- | --- |
|  | **N** | **HR** | **95% CI** | **p-value** | **HR** | **95% CI** | **p-value** |
| **Age at first line** | 686 | 1.03 | 1.02, 1.04 | <0.001 | 1.05 | 1.02, 1.08 | <0.001 |
| **Sex** | 686 |  |  |  |  |  |  |
| F |  | Ref. |  |  | Ref. |  |  |
| M |  | 1.51 | 1.23, 1.86 | <0.001 | 1.15 | 0.77, 1.70 | 0.5 |
| ***TP53* aberration** | 527 |  |  |  |  |  |  |
| No |  | Ref. |  |  | Ref. |  |  |
| Yes |  | 1.81 | 1.35, 2.42 | <0.001 | 1.41 | 0.86, 2.29 | 0.2 |
| **IGHV gene status** | 491 |  |  |  |  |  |  |
| Mutated |  | Ref. |  |  | Ref. |  |  |
| Unmutated |  | 2.12 | 1.63, 2.76 | <0.001 | 1.82 | 1.16, 2.87 | 0.010 |
| **Treatment categories** | 686 |  |  |  |  |  |  |
| BTKi |  | Ref. |  |  | Ref. |  |  |
| FCR/BR |  | 1.18 | 0.81, 1.71 | 0.4 | 1.08 | 0.54, 2.15 | 0.8 |
| Other |  | 1.95 | 1.29, 2.97 | 0.002 | 0.83 | 0.34, 2.08 | 0.7 |
| Other chemotherapy |  | 3.16 | 2.06, 4.82 | <0.001 | 0.84 | 0.33, 2.11 | 0.7 |
| Other CIT |  | 1.83 | 1.29, 2.61 | <0.001 | 1.06 | 0.54, 2.06 | 0.9 |
| Venetoclax-based |  | 0.34 | 0.12, 0.96 | 0.041 | 0.27 | 0.03, 2.06 | 0.2 |
| **Comorbidities at 1st-line treatment** | 608 |  |  |  |  |  |  |
| NO |  | Ref. |  |  | Ref. |  |  |
| YES |  | 1.21 | 0.92, 1.57 | 0.2 | 1.63 | 0.86, 3.08 | 0.13 |

CI = Confidence Interval, HR = Hazard Ratio, Ref. = reference category IGHV: Immunoglobulin heavy variable, BTKi: BTK inhibitor, FCR/BR: fludarabine, cyclophosphamide and rituximab/ bendamustine plus rituximab, CIT: chemoimmunotherapy
